# Quantifying the future risk of dengue under climate change in Japan

**DOI:** 10.3389/fpubh.2022.959312

**Published:** 2022-08-05

**Authors:** Katsuma Hayashi, Marie Fujimoto, Hiroshi Nishiura

**Affiliations:** School of Public Health, Kyoto University, Kyoto, Japan

**Keywords:** flavivirus, global warming, temperature, arbovirus, mathematical model, basic reproduction number

## Abstract

**Background:**

In metropolitan Tokyo in 2014, Japan experienced its first domestic dengue outbreak since 1945. The objective of the present study was to quantitatively assess the future risk of dengue in Japan using climate change scenarios in a high-resolution geospatial environment by building on a solid theory as a baseline in consideration of future adaptation strategies.

**Methods:**

Using climate change scenarios of the Model for Interdisciplinary Research on Climate version 6 (MIROC6), representative concentration pathway (RCP) 2.6, 4.5, and 8.5, we computed the daily average temperature and embedded this in the effective reproduction number of dengue, *R*(*T*), to calculate the extinction probability and interepidemic period across Japan.

**Results:**

In June and October, the *R*(*T*) with daily average temperature *T*, was <1 as in 2022; however, an elevation in temperature increased the number of days with *R*(*T*) >1 during these months under RCP8.5. The time period with a risk of dengue transmission gradually extended to late spring (April–May) and autumn (October–November). Under the RCP8.5 scenario in 2100, the possibility of no dengue-free months was revealed in part of southernmost Okinawa Prefecture, and the epidemic risk extended to the entire part of northernmost Hokkaido Prefecture.

**Conclusion:**

Each locality in Japan must formulate action plans in response to the presented scenarios. Our geographic analysis can help local governments to develop adaptation policies that include mosquito breeding site elimination, distribution of adulticides and larvicides, and elevated situation awareness to prevent transmission *via* bites from *Aedes* vectors.

## Introduction

Dengue fever is a mosquito-borne infectious disease found in most tropical and subtropical areas, which causes an estimated five million confirmed cases and several thousand deaths each year worldwide (1). Dengue is endemic in tropical areas where the disease is present throughout the year, as well as in other regions where the disease is not seen in the winter season and only sporadic minor outbreaks occur during warm and wet seasons *via* imported infections (2). Owing to climate change, dengue fever is expected to spread to previously unaffected areas (3). Dengue virus is transmitted by *Aedes albopictus* and *Aedes aegypti*, and these vector insects increase their activity in response to temperature (4). Many entomologic, virologic, and modeling studies have demonstrated that climate change would increase the impact of dengue across the globe (2, 3, 5), contributing to an elevated disease burden (6).

Climate change is a major public health concern. At its most recent conference, the Intergovernmental Panel on Climate Change established the goal of limiting the increase in global average temperature by the end of the twenty-first century to 1.5◦C (7). Forecasting the burden of dengue infections at the global level is essential; however, forecasting at the regional level is even more critical when considering adaptation policies (8). Currently, Japan is not a dengue-endemic country, and prior to the COVID-19 pandemic, ~50–300 confirmed imported dengue cases were reported annually in Japan (9). However, the years 2013–2014 were an exception. In 2013, a German tourist returning from Japan who had no recent history of travel to any dengue-endemic country was diagnosed with dengue fever upon her return to Germany. The possibility of autochthonous dengue transmission at a local level in Japan was suspected (10). Subsequently, in 2014, the first domestic outbreak since 1945 was reported in Tokyo, involving a total of 160 confirmed cases of dengue fever from August to October (11). However, there have been no subsequent reports of domestic infections as of June 1st, 2022 (12).

Numerous studies have predicted the increase in dengue owing to climate change in dengue-endemic countries (13). Most studies have involved time-series predictions using approaches such as autoregressive models (e.g., ARIMA) (14) or predictions using climate change parameters as variables in the effective reproduction number, i.e., the average number of secondary transmissions in humans produced by each primary case of human infection *via* a mosquito vector (15). In addition to mechanistic modeling studies that predict a future increase in the risk of dengue, more thorough spatiotemporal projections of dengue are needed, including regarding the probability of a major epidemic given dengue virus importation (16). We previously estimated the probability of an epidemic in Japan using the time-inhomogenous branching process, focusing on the risk around 2014 in Japan (17). Ishizaki recently developed a climate change projection model for devising adaptation policies in Japan by regularly updating climate change scenarios in the country. In that model, projected climatologic variables in fine spatial scale are produced through the year 2100 (18). Combining a stochastic model of dengue with climate change scenarios, it becomes possible to evaluate the probability and magnitude of future dengue epidemics in Japan, both spatially and temporally.

The objective of the present study was to quantitatively assess the risk of dengue in Japan based on climate change scenarios in a high-resolution geospatial environment, building on a solid theory as a baseline in consideration of future adaptation strategies.

## Materials and methods

### Data source for climate model

In this study, we used bias-corrected climate scenarios across Japan based on the cumulative distribution function-based downscaling method using the Climate Model Intercomparison Project Phase 6 (CMIP6) (18). The projection model is a bias-corrected climate scenario for a 1-km area of Japan (covering 377,449 geographic sites on the Japanese landmass) and is based on five global climate models [Model for Interdisciplinary Research on Climate version 6 (MIROC6), MRI-ESM2-0, ACCESS-CM2, IPSL-CM6A-LR, MPI-ESM1-2-HR] from CMIP6 with historical data, that offers scenarios of SSP1-representative concentration pathway (RCP)2.6, SSP2-RCP4.5, and SSP5-RCP8.5. The SSP1-RCP2.6, SSP2-RCP4.5, and SSP5-RCP8.5 scenarios are the climate change projection models proposed in the CMIP, classified according to carbon dioxide emissions and human socioeconomic activity (7). Historical data comprise a simulation model based on observations from 1900 to 2015. A modified version of Iizumi et al. (19) (2010, 2011, 2012, 2014, 2017) was applied as the bias correction method. This method is non-parametric, and error detection and correction are performed using the monthly cumulative density function. Daily data are available for eight variables (daily minimum, maximum, and average temperature, precipitation, total solar radiation, downward longwave radiation, wind speed, and relative humidity) for 1900–2100. Of the available scenarios, we specifically used the MIROC6 model, which was cooperatively developed by a Japanese modeling community to meticulously reflect the meteorological conditions in Japan (20). All data are available from the National Institute for Environmental website (18).

### Quantifying the effective reproduction number

The effective reproduction number of dengue virus, *R*(*T*), given temperature in degrees Celsius, *T*, can be written as follows (8, 21, 22):


(1) $$\begin{array}{l} R\left(T\right)=\frac{ma^{2}bc}{r\mu }e^{-\mu EIP}, \end{array}$$

where μ is the mosquito vector mortality rate, *r* is the human host recovery rate, *m* is the vector-to-host ratio, *a* is the vector bite rate, *b* is the transmission coefficient from human to vector, *c* is the transmission coefficient from vector to human, and EIP is the extrinsic incubation period. All seven variables appearing in Equastion 1 can be expressed as a function of mean temperature. The respective parameters *m* = 0.37, *r* = 0.2, and μ_A_ = 0.02 are taken from our previous study (17), where μ_A_ is the maximum adult mortality rate. The reproduction number *R*(*T*) takes the value 0 in the range *T* ≤ 14.6 and is an upward convex function in the range 14.61 < *T* <40.4. The analytical solution for which *R*(*T*) = 0 at 14.61 < *T* <40.4 is *T* = 28.5 (i.e. the derivative of *R*(*T*) being zero), where the maximum value is *R*(*T* = 28.5) = 3.38, indicating that if the average temperature is too high, the value of the reproduction number in this particular model decreases; a past study supports this phenomena (21). For simplicity, in the present study, we aimed to avoid underestimation of the risk; thus, we did not adopt the controversial decrease in *R*(*T*) over 28.5◦C. In other words, we defined *R*(*T*) as:


(2) $$R\left(T\right)=\{\begin{array}{c} \frac{ma^{2}bc}{r\mu }\exp\left(-\mu EIP\right),\;for\;T\;<28.5\\ 3.38,\;for\;28.5\leq T \end{array}.$$

### Projection of dengue risk

#### Future temperature

Daily data are available for projected average temperatures up to the year 2100. The method of uncertainty representation in this study was based on a state-space model for the time series of average temperatures. In particular, when representing the long-term time series, a specific day in the year (e.g., July 1) was selected from 1990 to 2100, and we considered these data to be observed values *y*_t_. The expected value of the average temperature is α_t_, excluding the observation error ε_*t*_with mean 0 and variance *H*. In other words:


(3) $$\begin{array}{lll} y_{t} & = & \alpha_{t}+\varepsilon_{t},{\;\varepsilon }_{t}\sim N\left(0,H\right),\;t=1,2,\ldots ,n, \end{array}$$

(4) $$\begin{array}{lll} \alpha_{t+1} & = & \;\alpha_{t}+\eta_{t,}\;\eta_{t,}\sim N\left(0,Q\right),\;t=1,2,\ldots ,n-1, \end{array}$$

where η_*t*_ is the state disturbance term for the expected value α_*t*_, assuming mean 0 and variance Q. We also assumed that the initial state is


(5) $$\begin{array}{l} \alpha_{1}=N\left(\alpha_{1},P_{1}\right), \end{array}$$

and that the initial state follows a normal distribution according to We also assumed that ε_*t*_, η_*t*_, α_1_ are all independent of each other. Using Equations 3–5, the expected value of α_*t*_ and the 95% confidence interval (CI) of α_*t*_ in the temperature prediction model are estimated using the Kalman filtering and smoothing algorithm. In this study, temperatures were assumed to vary independently in the geospatial environment to reduce computational complexity. The estimation was performed using the R package “KFAS” (22, 23). The upper CI for the reproduction number was obtained by substituting 2.5th and 97.5th percentile of the CI of temperature in Equation 2.

#### Extinction probability of dengue

The extinction probability of dengue was computed using a branching process model (24–26). Assuming a negative binomially distributed offspring distribution with *R*(*T*) as the mean of the offspring distribution and *k* as the dispersion parameter, the probability *q*_*ti*_ that an infected person will not produce any secondary cases (that is, the extinction probability at time *t* in mesh *i*) was formulated as follows:


(6) $$\begin{array}{l} q_{ti}=\frac{1}{{\left(1+\frac{R_{ti}}{k}\left(1-q_{ti}\right)\right)}^{k}}\;. \end{array}$$

For the computation presented in the main text, we assumed that the dispersion parameter *k* → 1, i.e., a special case of the negative binominal distribution approximating the geometric distribution. We also examined the case when *k* = 10, i.e., the distribution is that is close to the Poisson distribution (*k* → ∞), and the case when *k* = 0.1, i.e., the distribution that has a long tail. The extinction probability here is the conditional probability of extinction given that a single infectious imported case entered a specific geographic mesh.

#### Interepidemic period

We calculated the reproduction number and extinction probability on a daily basis through 2100, and we counted the number of days in a year in which the extinction probability is 1 (i.e., *R* ≤ 1) and defined the total number of days without an outbreak as the interepidemic period (IEP) (27). During the IEP, theoretically, no large-scale epidemic will occur no matter how many infected people enter the country and whether any infection declines to extinction. Therefore, virtually no countermeasures need to be implemented during the IEP. The time series data of IEP from 1990 to 2100 were visualized, and the 95% CIs of the observed values are calculated using the KFAS package.

#### Risk mapping

Geographic distributions of the extinction probability and IEP were examined across Japan. Temperature and effective reproduction number were modeled for each geographic mesh *i*, mechanistically and thus independently, implying that our model assumed that a dengue outbreak within a single geographic mesh did not affect the risk in neighboring areas.

## Results

### Temporal distribution of daily average temperature

**Figure 1:**
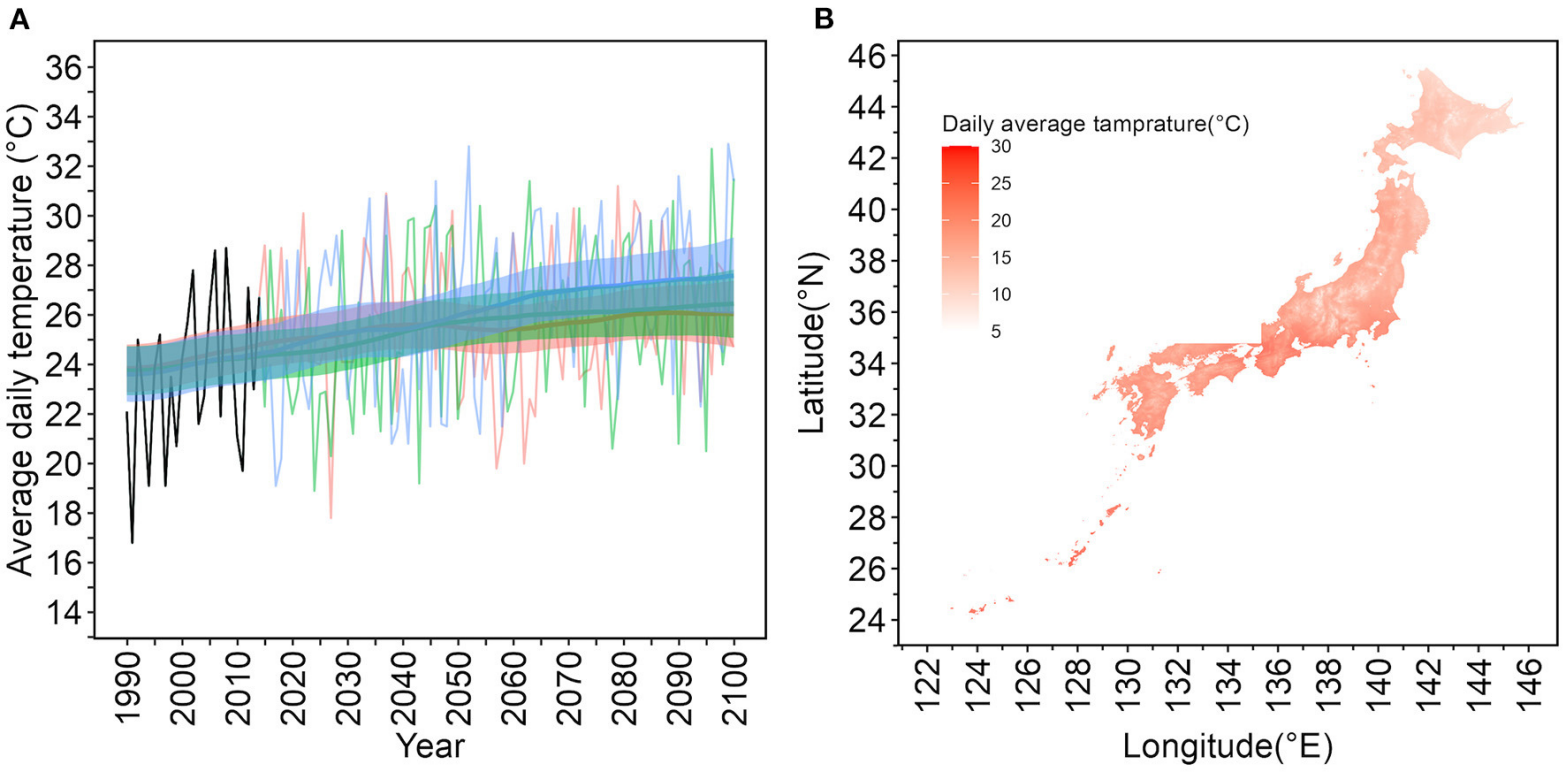
Daily average temperatures on July 1 from 1990 to 2100 in Tokyo based on the Model for Interdisciplinary Research on Climate version 6 (MIROC6) and a geographic snapshot of daily average temperatures in Japan. **(A)** Temporal distribution of daily average temperatures on July 1 in the specific mesh containing 35.6716◦N, 139.6967◦E, where Yoyogi Park in Tokyo is located. The years from 1990 to 2015 are historical data and reconstructed estimates based on observations. The MIROC6 was used to predict the period from 2016 to 2100. The oscillating lines are the model predictions. Thick lines are expected values estimated using Kalman filtering and smoothing. The shaded upper and lower lines are 95% confidence intervals of the expected values. Red lines represent the representative concentration pathway (RCP) 2.6 scenario, green the RCP4.5 scenario, and blue the RCP8.5 scenario. **(B)** Geographic snapshot of the average temperature on July 1, 2030 in Japan. The darker the red color, the higher the temperature. Japan is in the northern hemisphere, and temperatures are lower in the north. The snapshot shown is for July 1, during the summer season; however, even on the same day, there is a temperature difference of more than 20◦C within Japan.

Figure 1A illustrates the daily average temperature for a specific geographic mesh in Tokyo that contains Yoyogi Park, from 1990 to 2100, on July 1. From 2016 to 2100, RCP2.4, 4.5, 8.5 scenarios based on the MIROC6 model were used. The mesh information for Yoyogi Park in Tokyo is located at latitude 35.6716◦ N and longitude 139.6967◦. The figure displays the 95% CIs of mean values using Kalman filtering and smoothing. Figure 1B depicts a snapshot of the daily average temperature on July 1, 2030. Japan has a long north–south axis, and there is a difference of more than 20◦C on the same day between Hokkaido, the northernmost prefecture, and Okinawa, the southernmost prefecture, which belongs to a subtropical zone.

### Effective reproduction number in Tokyo

**Figure 2:**
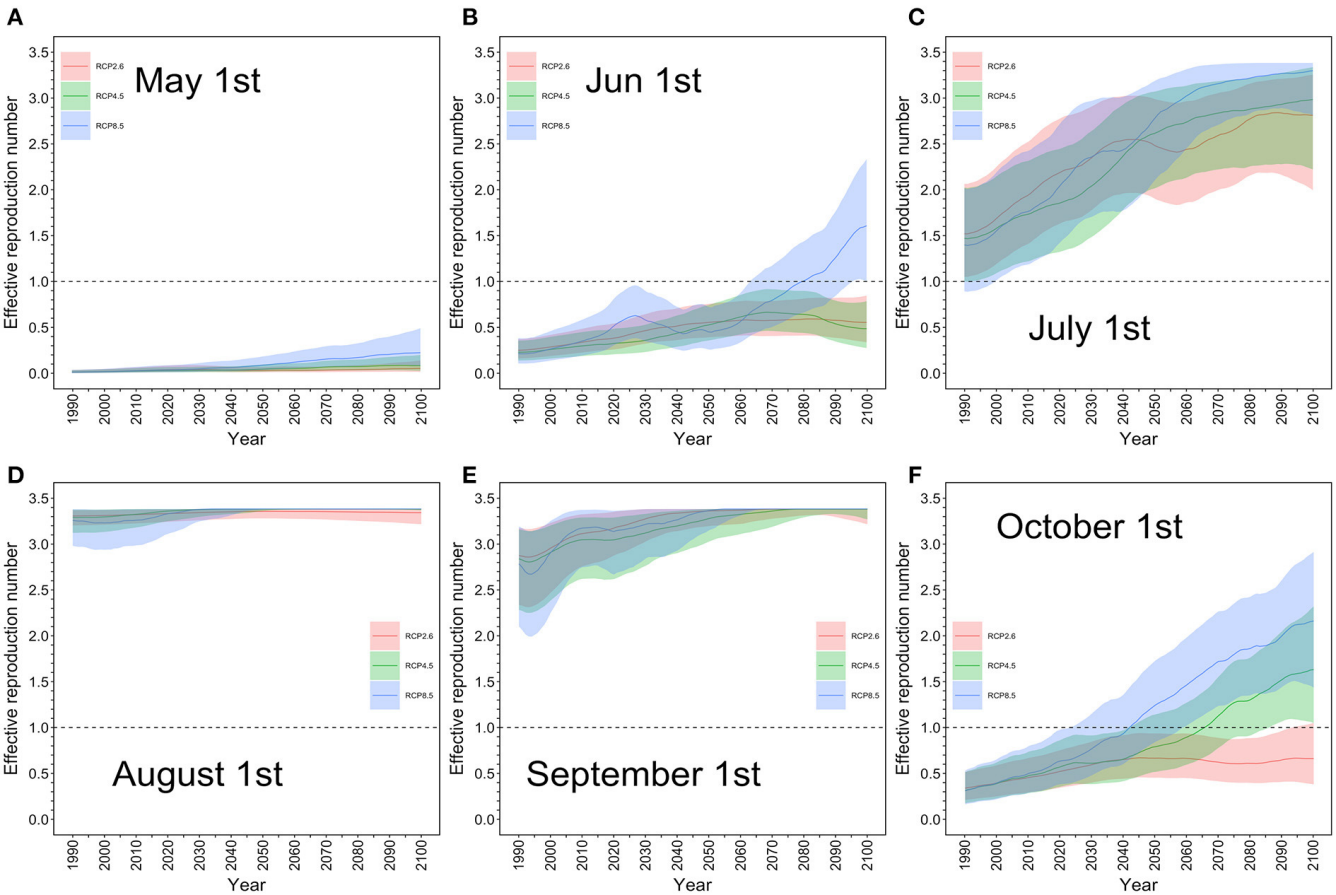
Projected effective reproduction number for each RCP scenario in Tokyo: May–October. The estimated effective reproduction number from May 1 to October 1 based on the MIROC-6 model is shown. The thick line represents the expected value, and the upper and lower shaded areas are the effective reproduction number with the 95% confidence interval based on the 95% confidence interval of daily average temperature. **(A)** Red is the RCP 2.6 scenario, green is the RCP 4.5 scenario, and blue is the RCP 8.5 scenario; **(A)** May 1, **(B)** June 1, **(C)** July 1, **(D)** August 1, **(E)** September 1, and **(F)** October 1. The maximum value of the effective reproduction number was 3.4 in the model, and there was little difference in the impact between the scenarios for August and September, the summer season in Japan. Even in the most pessimistic scenario, RCP8.5, the effective reproduction number was <1 on May 1, indicating that the risk of a dengue epidemic was low in Tokyo. However, in July, the effective reproduction number steadily rose to above 1 in all scenarios whereas in June and October, the value was <1, as in 2022.

The effective reproduction number *R*(*T*) in Tokyo was calculated using temperature *T* as an input variable. Figure 2 shows the predicted *R*(*T*) in the MIROC-6 model on May 1, June 1, July 1, August 1, September 1, and October 1 from 1990 to 2100 under the selected RCP scenarios. Little impact was seen in August and September, the warmest summer months, as the ceiling of *R*(*T*) was reached. During these months, the risk of a dengue epidemic remained high in Tokyo. In July, the *R*(*T*) increased as a function of time, steadily rising to above 1. In June and October, the *R*(*T*) was <1, as in 2022, but elevation of the *R*(*T*) during these months was more pronounced under RCP8.5 than under the other two scenarios, considerably influenced by a marked increase in temperature. Our estimate in July, Tokyo, 2014 was estimated to be up to 3.4, while our published estimate that analyzed the actual outbreak has shown that *R*(*T*) was overall on the order of 2–5 (28).

### Extinction probability

**Figure 3:**
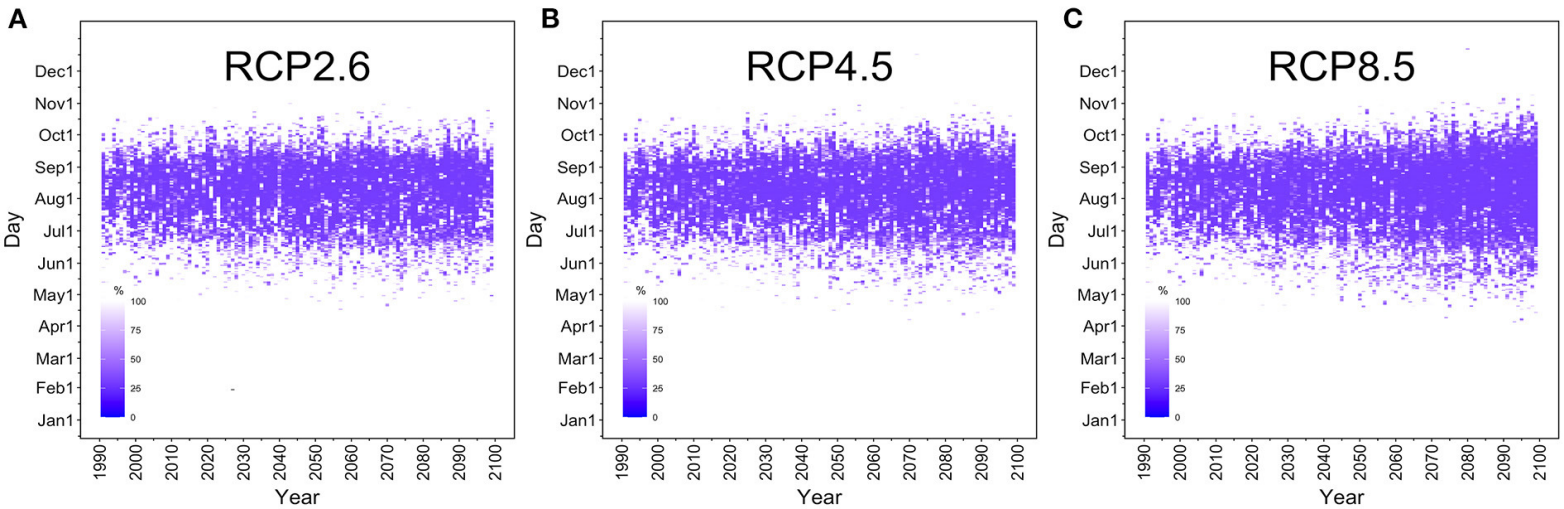
Time-dependent changes in the dengue extinction probability in Tokyo. The horizontal axis shows the years 1990 to 2100. The vertical axis measures the period from January 1 to December 31. The density of the purple area reflects the daily extinction probability in Tokyo's Yoyogi Park. The extinction probability at 100% is shown in white. **(A)** The RCP2.6 scenario, **(B)** RCP4.5 scenario, and **(C)** RCP8.5 scenario. Under the most optimistic RCP2.6 scenario, there is little difference between the years 2022 and 2100. However, under the RCP8.5 scenario, the risk of a large-scale dengue epidemic is gradually extended not only during the summer (June–September) but also in the autumn and spring to include November and April.

Figure 3 shows the temporal distribution of the extinction probability in the selected mesh in Tokyo. The optimistic RCP2.6 scenario showed no marked increase in the number of days with *R*(*T*) >1 through 2100. However, under the RCP8.5 scenario, our model showed that the risk period of dengue transmission was markedly lengthened over the course of time, gradually extending to late spring (April–May) and autumn (October–November). Supplementary Figure 1 shows the extinction probabilities for various dispersion parameters. When *k* is small, that is, when the tail of the distribution is long, the extinction probability becomes large even for the same effective reproduction number, indicating that the epidemic easily declines to extinction spontaneously. If the distribution is close to the Poisson distribution (i.e. large *k*-value), the probability of a large epidemic from one infected case increases. The early period estimates in 1990 showed that *R*(*T*) was smaller than or at least close to the value of 1, endorsing the fact that Japan was dengue free for 70 years by 2014 outbreak.

### Interepidemic periods

**Figure 4:**
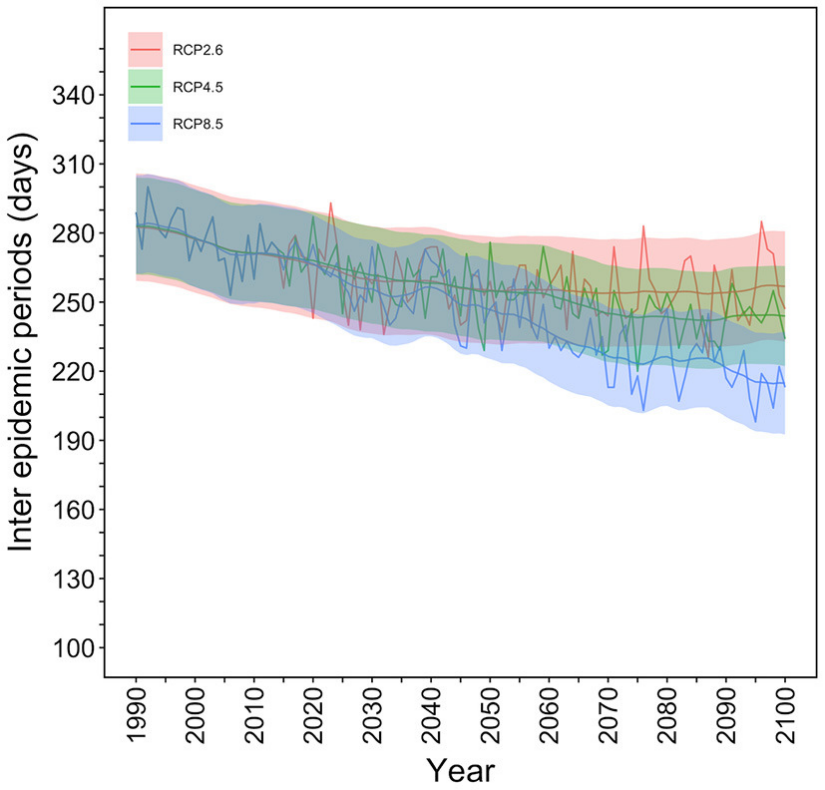
Interepidemic periods in Tokyo for each RCP scenario. The number of days in a year when the extinction probability is 100% were counted and defined as the interepidemic period (IEP). The temporal distribution of the IEP for the selected mesh, which contains Yoyogi Park in Tokyo, is shown. The thick line shows the expected IEP values, and the upper and lower shaded areas are 95% confidence intervals (CIs), including the observation error calculated using Kalman filtering and smoothing. In 1990, IEP values were 280 (95% CI: 260–302) days per year, but this decreased by ~2 months to 220 days (95% CI: 190–240) under RCP8.5 scenario in the year 2100.

The temporal distribution of the IEPs for the selected mesh in Tokyo is shown in Figure 4. Under the RCP8.5 scenario, a total of 280 (95% CI: 260–302) days were considered the IEP in 1990 whereas only 220 (95% CI: 190–240) days were considered the IEP in the year 2100. Namely, under the RCP8.5 scenario, the dengue-free period was shortened by 2 months. Under the RCP2.6 scenario, the IEP in 2100 was calculated to be 260 (95% CI: 240–290) days, and the dengue–free period would be shortened by 20 days in comparison with the year 1990.

### Snapshot of extinction probability and days of IEP

**Figure 5:**
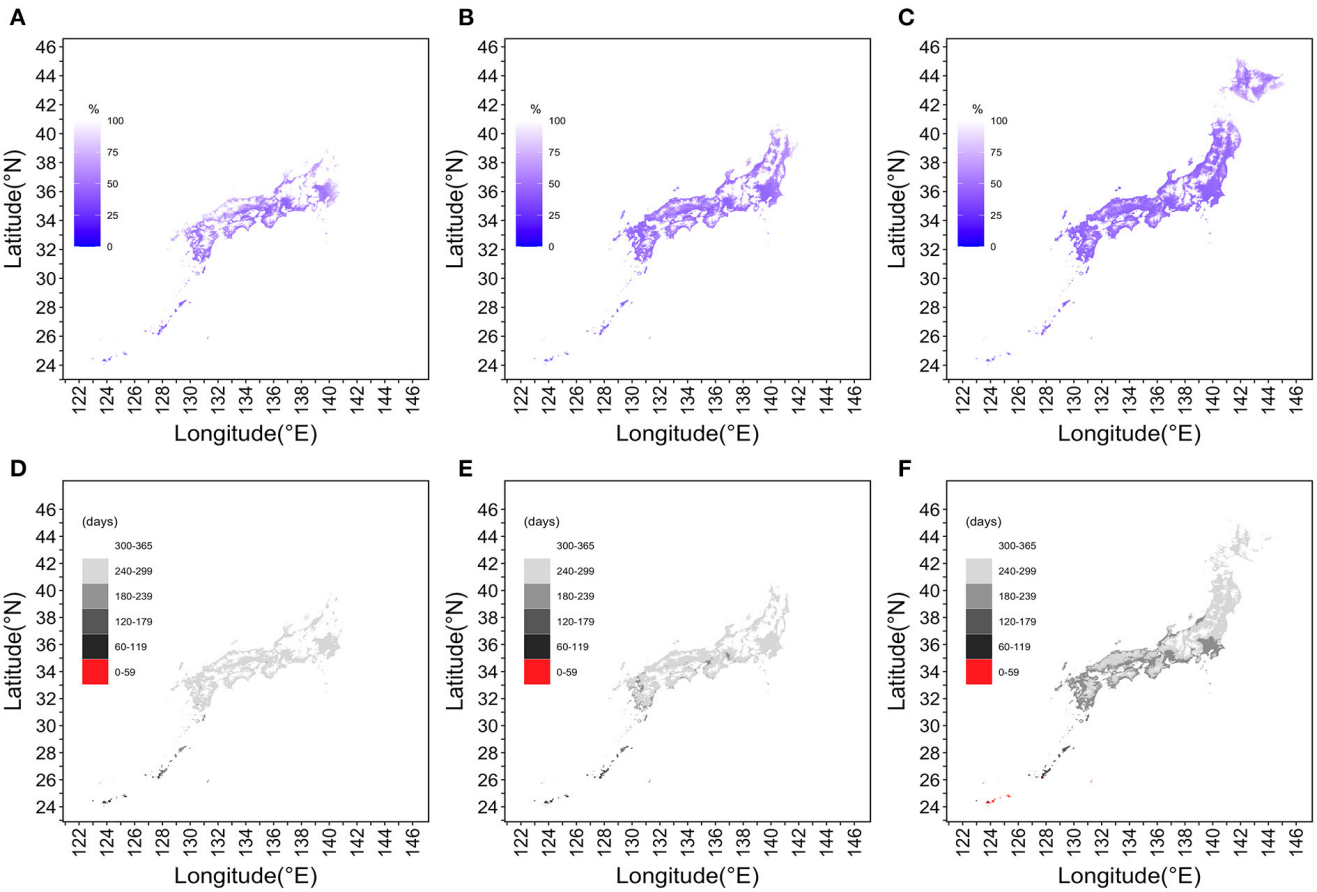
Snapshot of the extinction probability and interepidemic period across Japan in 2030, 2050, and 2100 under the RCP8.5 scenario. **(A,D)** depict the year 2030, **(B,E)** the year 2050, and **(C,F)** the year 2100. **(A–C)** The probability of extinction in the Tohoku region and Hokkaido on July 1, 2030. However, in 2050, the epidemic risk area had moved northward and from coastal to inland areas. Although merely a snapshot, our mapping shows that the epidemic risk is extended over the entire Hokkaido region by 2100. In **(D–F)**, IEPs are divided into six discrete categories and Japan is mapped at a resolution of 1 km^2^. The darker the gray color, the shorter the IEP and the longer the period of dengue epidemic risk. The RCP8.5 scenario for the year 2100 shows that parts of the Okinawa Islands will have IEPs of fewer than 60 days, and southwestern Hokkaido will have dengue transmission risk periods longer than 60 days.

Figure 5 shows how the extinction probability and IEP behaved over space and time. Data for 377,449 geographic locations (i.e., for each 1 km^2^ mesh) were examined, excluding ocean areas. In Figures 5A–C, the extinction probability on July 1, 2030 was calculated to be 0% in Tohoku and Hokkaido. However, the epidemic risk area had moved northward over time; the geographic areas with a risk for dengue transmission had also spread from coastal to inland areas in 2050. By the year 2100, the epidemic risk extended to all of northernmost Hokkaido Prefecture. Figures 5D–F shows the IEPs divided into six discrete categories and maps with the estimated values, with a geographic resolution of 1 km^2^. Under the RCP8.5 scenario, a part of southernmost Okinawa Prefecture would have an IEP of fewer than 60 days by the year 2100. In southwest Hokkaido, the yearly period with a high risk of dengue transmission continued for more than 60 days in the year 2100.

## Discussion

In the present study, we explored the future risk of dengue under climate change scenarios over time and space in Japan, a temperate zone country. Using MIROC6 climate change scenarios RCP2.6, 4.5, and 8.5, we computed the daily average temperature and embedded this in the effective reproduction number of dengue to calculate the extinction probability and IEP. In June and October, the effective reproduction number of dengue, *R*(*T*), was <1, as in 2022; however, an elevation of temperature increased the number of days with an *R*(*T*) >1 during these months under RCP8.5. The time period with a risk of dengue transmission gradually extended into late spring (April–May) and autumn (October–November). Under the RCP8.5 scenario in 2100, a part of southernmost Okinawa Prefecture exhibited the possibility of no dengue–free months, and the epidemic risk extended to the entire part of northernmost Hokkaido Prefecture.

To the best of our knowledge, the present study was the first to assess the long-term risk of dengue in Japan under climate change, specifically using projected RCP scenarios and high-resolution geospatial mesh data. Under the most pessimistic RCP8.5 scenario, dengue risk could be present in Tokyo during May and November in 2100. For Okinawa Prefecture, the warmest region of Japan, our results indicated possible year-round dengue occurrence by 2100. Even in northernmost Hokkaido, the dengue risk would develop into a serious one. Overall, an increase in the average temperature of 2–4◦C would considerably change the epidemiology of dengue and increase the risk across Japan. The epidemic during 2014 in metropolitan Tokyo should serve as a warning signal of ongoing elevation in the risk of dengue transmission.

It is a scientific fact that climate change will increase the average global temperature (29). In this study, we visually depicted the risk of dengue epidemics in a geographic segment with calculation of the extinction probability. On the basis of such quantified scenarios, both mitigation and adaptation strategies must be seriously considered. In particular, our geographic analysis can help local governments to formulate adaptation policies that include standing water elimination, distribution of adulticides and larvicides, and elevated situation awareness regarding how to prevent bites from *Aedes* mosquitos (e.g., wearing long sleeves in the summer, using repellents and avoiding spending time in areas with abundant mosquito populations). The take-home message of the present study is that each locality must formulate action plans in response to the presented scenarios.

The question arises of when local authorities should begin implementing countermeasures against dengue. We believe that our calculation of the IEP will be of assistance in developing seasonal interventions. When the risk of dengue becomes non-negligible in warmer seasons, local authorities should begin to recommend the use of insect repellents, such as those containing DEET, for the local population (30). Additionally, more stringent countermeasures, e.g., the use of insecticides in public areas and intensive standing water management, can be planned for areas with a high epidemic risk. By adding evidence to existing studies reporting on the habitat areas of *Aedes albopictus* in Japan (31), we believe that the present study findings inform concrete ideas according to quantified risk that will directly lead to appropriate local countermeasures. It should be noted that this study calculated the conditional probability of extinction given an introduction of single infected individual. Thus, assuming that the number of independently importing cases is *n*, the probability of outbreak can be calculated as $1-{q_{ti}}^{n}$. This study can be further extended to account for the number of importations by additionally using the number of imported cases, e.g., by estimating the number of introductions near airport or in touristic spot where many foreign visitors gather (32).

An important role of the present work among studies on the future dengue risk in Japan is to offer scenarios for further analysis. Whereas, temperature was indeed useful in capturing the forthcoming elevated dengue risk across Japan, future interventions must account for additional climatologic elements, especially precipitation. One key to dealing with dengue infections is eliminating mosquito breeding sites and larval habitats: i.e., a container-based model (33). Mosquito larvae require pooled water such as that accumulated in abandoned tires and gutters that have not been cleared or treated insecticides (34). A correlation between precipitation and mosquito larval populations has been observed in many studies. Theoretically, if standing water sources are effectively eliminated, the impact of precipitation on dengue infections will be small (35). Because quantification of *Aedes* breeding sites in Japan is inadequate compared with countries that are heavily affected by dengue infections, such as those in Southeast Asia and Latin America (33), in the present study, we did not include precipitation as a parameter. Moreover, precipitation has an essential interrelationship with temperature (36). However, caution is needed in extrapolating the findings from tropical countries to Japan, which has four distinct seasons with only hot and humid summers.

The present study involved the following limitations. First, the proposed model used only daily average temperature as the input parameter. In addition to the average temperature, the climate variability parameters associated with the magnitude of dengue epidemics include maximum temperature (37), minimum temperature (38), daily temperature range (4), precipitation (36), humidity (39), and consecutive rainy days (36). Although there are many studies on the interdependence of these parameters in time-series models, no model fully accounts for the dependence among variables and mechanistically incorporates them into the *R*(*T*). At least, we have verified in advance that published studies on the relationship between temperature and the dengue outbreak have shown that daily average temperature was most strongly correlated with the incidence of dengue (40). Further research is deemed essential to identify a model that can more accurately calculate the extinction probability for Japan. Second, the proposed model did not explicitly account for geospatial dependencies, assuming that the mechanistic process was conditionally independent, with a single geographic mesh. The imposed assumption may be valid while the risk of dengue remains small, but when the number of cases increases or when conditions such as airports or tourist attractions can be taken into consideration explicitly, geographically dependent models need to be considered (41). That is, accounting for geographic dependence is not necessarily required as long as the epidemic risk is maintained small. However, once the major epidemic becomes unavoidable, each geographic unit starts to interact from each other, elevating the overall risk of epidemic. Addressing geospatial dependence would be then vital. Third, caution must be exercised as to the method used to quantify uncertainty. In the present study, we used Kalman filtering and smoothing for model predictions, representing uncertainty with respect to climate change scenarios. Apart from the included uncertainty, we performed deterministic calculations of the effective reproduction number. One parameter for *R*(*T*), *m*, which represents mosquito abundance, was fixed at 0.37, an estimate for the 2014 outbreak in Yoyogi Park, Tokyo (17). This value would vary according to the volume of standing water, precipitation, and local population density. Further studies are deemed essential for a rigorous assessment of these uncertainties.

Despite the above limitations, in this study, we successfully mapped the extinction probability of dengue fever in Japan temporally and geographically using the branching process model. The present results lay a foundation for future studies to accurately calculate the dengue epidemic risk and develop appropriate adaptation measures.

## Data availability statement

The original contributions presented in the study are included in the article/Supplementary material, further inquiries can be directed to the corresponding author.

## Author contributions

HN conceived the study idea. KH analyzed the empirical datasets. KH and HN reviewed the results and drafted manuscript. All authors provided comments and approved the final version of the manuscript.

## Funding

KH received funding from the Japan Society for the Promotion of Science (JSPS) KAKENHI (20K18953) and The Health Care Science Institute (IKEN). This study was mainly supported by the Environment Research and Technology Development Fund (JPMEERF20S11804) of the Environmental Restoration and Conservation Agency of Japan. HN received funding from Health and Labor Sciences Research Grants (20CA2024, 20HA2007, 21HB1002, and 21HA2016), the Japan Agency for Medical Research and Development (JP20fk0108140, JP20fk0108535, and JP21fk0108612), the JSPS KAKENHI (21H03198), and the Japan Science and Technology Agency SICORP program (JPMJSC20U3 and JPMJSC2105). The funders had no role in the study design, data collection and analysis, decision to publish, or preparation of the manuscript.

## Conflict of interest

The authors declare that the research was conducted in the absence of any commercial or financial relationships that could be construed as a potential conflict of interest.

## Publisher's note

All claims expressed in this article are solely those of the authors and do not necessarily represent those of their affiliated organizations, or those of the publisher, the editors and the reviewers. Any product that may be evaluated in this article, or claim that may be made by its manufacturer, is not guaranteed or endorsed by the publisher.

## Supplementary material

Supplementary figure 1 Extinction probability with different dispersion parameters. **(A–C)** Estimate on May 1; **(D–F)** show estimate on June 1; **(G–I)** show estimate on July 1; **(J–L)** show estimate on August 1; **(M–O)** show estimate on September 1; **(P–R)** show estimate on October 1. Extinction probabilities are shown from 1990 to 2100. The left panels show results with dispersion parameter *k* = 0.1 in equation (2), the middle panels with *k* = 1, and the right panels show results with *k* = 10. The solid red line indicates the RCP2.6 scenario, green indicates the RCP4.5 scenario, and blue indicates the RCP8.5 scenario. Confidence intervals are calculated by substituting the upper and lower limits of the 95% confidence interval for the mean temperature using the variance estimated from equations (3, 4) into (6).
