# Supplementary figures and images for: Quantifying the future risk of dengue under climate change in Japan
Source: Front Public Health. 2022 Aug 5;10:959312. doi: 10.3389/fpubh.2022.959312 (PMC9389175; doi:10.3389/fpubh.2022.959312)

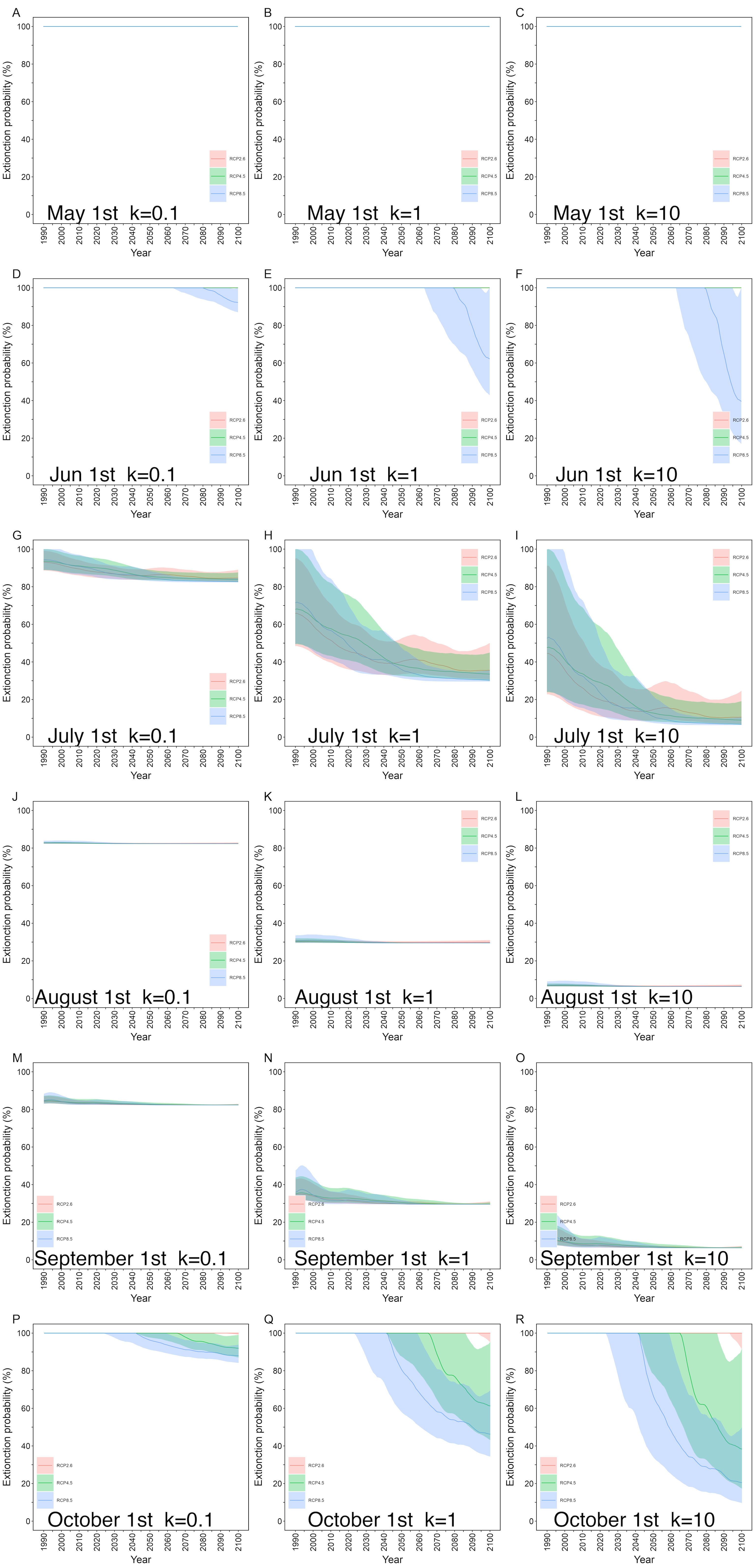

Supplement: Supplementary figure 1 — Extinction probability with different dispersion parameters. (A–C) Estimate on May 1; (D–F) show estimate on June 1; (G–I) show estimate on July 1; (J–L) show estimate on August 1; (M–O) show estimate on September 1; (P–R) show estimate on October 1. Extinction probabilities are shown from 1990 to 2100. The left panels show results with dispersion parameter k = 0.1 in equation (2), the middle panels with k = 1, and the right panels show results with k = 10. The solid red line indicates the RCP2.6 scenario, green indicates the RCP4.5 scenario, and blue indicates the RCP8.5 scenario. Confidence intervals are calculated by substituting the upper and lower limits of the 95% confidence interval for the mean temperature using the variance estimated from equations (3, 4) into (6). [file Image_1.TIFF]
